# Trends and outcomes in community-onset and hospital-onset *Staphylococcus bacteremia* among hospitals in the United States from 2015 to 2020

**DOI:** 10.1017/ash.2024.402

**Published:** 2024-09-16

**Authors:** Takaaki Kobayashi, ChinEn Ai, Molly Jung, Jorge L. Salinas, Kalvin C. Yu

**Affiliations:** 1 Carver College of Medicine, University of Iowa, Iowa City, IA, USA; 2 Medical & Scientific Affairs, Becton, Dickinson and Company, Franklin Lakes, NJ, USA; 3 Division of Infectious Diseases, Department of Internal Medicine, Stanford University, Stanford, CA, USA

## Abstract

**Background:**

We investigated trends in *Staphylococcus aureus* (staph) bacteremia incidence stratified by methicillin susceptibility (methicillin-susceptible *S. aureus* [MSSA] vs. methicillin-resistant *S. aureus* [MRSA]) and onset designation (community-onset [CO] vs. hospital-onset [HO]).

**Methods:**

We evaluated the microbiological data among adult patients who were admitted to 267 acute-care hospitals during October 1, 2015, to February 28, 2020. Using a subset of data from 41 acute-care hospitals, we conducted a retrospective cohort study to assess patient demographics, characteristics, mortality, length of stay, and costs. We also conducted a case-control study between those with and without staph bacteremia.

**Results:**

The incidence of MSSA bacteremia significantly increased from 2.43 per 1,000 admissions to 2.87 per 1,000 admissions (estimate=0.0047, *P*-value=.0006). The incidence of MRSA significantly increased from 2.11 per 1,000 admissions to 2.42 per 1,000 admissions (estimate=0.0126, *P*-value <.0001). While the incidence of CO MSSA and CO MRSA demonstrated a significant increase (p=0.0023, and p < 0.0001), the incidence of HO MSSA and HO MRSA did not significantly change (p=0.2795 and p < 0.4464). Compared to those without staph bacteremia, mortality, length of stay, and total cost were significantly higher in those with staph bacteremia, regardless of methicillin susceptibility or onset designation.

**Conclusion:**

The increasing incidence of CO MSSA and MRSA bacteremia might suggest the necessity for dedicated infection control measures and interventions for community members colonized with or at risk of acquiring *Staphylococcus aureus*.

## Background

*Staphylococcus aureus* (staph) bacteremia holds substantial clinical significance and represents a public health concern, being a leading cause of both community-acquired and hospital-acquired bacteremia.^1,2^ Staph bacteremia has a mortality rate up to 30% and often requires prolonged antimicrobial therapy.^3,4^ Methicillin-resistant *S. aureus* (MRSA) is recognized as an enduring threat and has been classified as a high-priority pathogen by the World Health Organization.^5^ However, the incidence of methicillin-susceptible *S. aureus* (MSSA) and MRSA bacteremia has shown temporal fluctuations, with varying trends observed across different geographic regions.^6–10^ Earlier research suggests that MRSA tends to lead to additional infections rather than replacing those caused by MSSA, and trends in MRSA and MSSA may diverge based on infection site and population characteristics.^6,11–13^

Between 2005 and 2018, data extracted from the European Antimicrobial Resistance Surveillance Network revealed a statistically significant upswing in the incidence of staph bacteremia within the European Union and European Economic Area (EU/EEA).^9^ Notably, this surge primarily stemmed from an increase in MSSA bacteremia cases, while the incidence and proportions of MRSA bacteremia diminished throughout the same timeframe within the EU/EEA.

Conversely, in the United States, analysis of electronic health records from over 400 acute-care hospitals, in conjunction with population-based surveillance data from the Centers for Disease Control (CDC) and Prevention’s Emerging Infections Program (EIP), showed a national reduction in MRSA bacteremia in healthcare settings, averaging approximately 17% annually between 2005 and 2012.^6^ Nonetheless, a noteworthy deceleration in these reductions was observed from 2012 to 2017, raising concerns.^6^ The same author also noted that hospital-onset (HO) MSSA bacteremia remained relatively stable, while community-onset (CO) MSSA bacteremia infections showed a slight increase from 2012 to 2017. Another study in the United States reported a sharp decline in staph infections, including bloodstream and non-bloodstream infections, from 2005 to 2017 in U.S. Department of Veterans Affairs medical centers, with most reductions attributed to decreases in MRSA infections.^14^ Moreover, a population-based study in Minnesota examining the incidence of monomicrobial staph bacteremia between 2006 and 2020 demonstrated that there was no apparent temporal trend over 15 years with a stable incidence.^15^ While some studies have examined the trend of staph bacteremia, research on the trends in CO and HO staph bacteremia is limited, especially in the United States. This research gap may become increasingly clinically relevant with the forthcoming launch of volunteer reporting of Hospital Onset Bacteremia & Fungemia (HOB) events by the CDC/National Healthcare Safety Network (NHSN) in the United States.^16^

Our primary objective is to elucidate trends in staph bacteremia incidence, categorized by methicillin susceptibility (MRSA versus MSSA) and onset designation (CO vs. HO), in the United States. Additionally, we assess patient demographics, characteristics, mortality, length of stay (LOS), and costs among patients with staph bacteremia and compare them to those without staph bacteremia.

## Methods

### Study design and population

We conducted an epidemiological study, evaluating the microbiological data among adult patients, aged 18 years or older, who were admitted to any of the 267 acute-care hospitals assessed in the BD Insights and Research Database (Becton Dickinson, Franklin Lakes, NJ) during the period spanning from October 1, 2015, to February 28, 2020. The hospital demographic distribution of 267 acute-care hospitals is shown in Supplemental Table 1a. This database incorporates electronically recorded laboratory results, pharmacy records, patient demographics, administrative data, as well as admission, discharge, and transfer data.^17–19^

As claims data were available for a subset of facilities (41 out of the 267 acute-care hospitals), we conducted a retrospective cohort study limited to those hospitals to assess mortality rates, length of hospital stays, and associated costs. The hospital demographic distribution of 41 acute-care hospitals is shown in Supplemental Table 1b. Additionally, we conducted a case-control study comparing individuals with and without staph bacteremia to investigate differences in LOS, mortality, 30-day readmission, and total cost. This study received approval as a limited retrospective dataset for epidemiological analyses and was granted an exemption from the requirement for consent by the New England/WCG Institutional Review Board and Human Subjects Research Committee (Wellesley, MA). The study was executed in strict accordance with the regulatory standards set forth by the Health Insurance Portability and Accountability Act.

### Outcomes and definitions

In our analyses, we considered the day of admission as “day 1.” An HO MSSA and HO MRSA was defined as the first positive blood culture with MSSA or MRSA, which begins on day 4 or later of the hospital stay, consistent with the HOB definition planned by the NHSN at the time of writing. CO MSSA and CO MRSA were defined as a first positive blood culture with a MSSA or MRSA as defined by the CDC within the first 3 days of hospital admission.^19^ The control group was defined as inpatient admissions with at least one blood specimen collection but without any positive staph bacteremia identified. Therefore, the control group included those with negative blood cultures and those with positive blood cultures for bacteria other than *Staphylococcus aureus*.

Variables assessed include age, gender, year of diagnosis, payor class, presence of intensive care (ICU) admission, LOS in the ICU, Acute Laboratory Risk of Mortality Score (ALaRMS, an EHR-derived comorbidity measure),^20^ total LOS in the hospital, payor type, readmission within 30 days, and in-hospital mortality, and hospital-level variables (hospital staffed bed size, hospital teaching status, hospital urbanicity, hospital region (CDC geographics census division [9 divisions]).^21^ We noted whether patients with staph bacteremia had any of the following six infectious disease diagnoses commonly associated with staph bacteremia: central line-associated bloodstream infection, infective endocarditis, musculoskeletal infections, phlebitis and thrombophlebitis, pneumonia, and skin and soft tissue infection.^22^The International Classification of Diseases, 10th Revision (ICD-10 codes) were employed to identify these six diagnoses (please refer to Supplemental Table 2 for code details). Rural vs. urban patient residence was determined using Rural Urban Commuting Area codes linked to ZIP codes.^23^

### Statistical analysis

For the epidemiological study, we plotted the incidence of CO staph bacteremia stratified by methicillin susceptibility over time. The incidence of CO MSSA/MRSA was computed by dividing the number of CO MSSA/MRSA admissions by the total count of inpatient admissions, multiplied by 1000. As the risk of HO bacteremia may vary with the duration of patient admission, we also produced separate plots for HO MSSA/MRSA. The incidence of HO MSSA/MRSA was determined by dividing the number of HO MSSA/MRSA admissions by the total patient days at risk, multiplied by 1000. If admissions included CO MRSA/MSSA cases, the patient days at risk were excluded from the denominator of HO MRSA/MSSA. Patient days at risk were defined as the number of days from the commencement of admission to the occurrence of the event, excluding days following the fulfillment of one of the infection criteria. To assess the trend in the incidence rates for total MSSA, total MRSA, CO MSSA, HO MSSA, CO MRSA, and HO MRSA, we employed the generalized estimating equations technique using the Poisson distribution. To quantify the change in the incidence over time, we used Poisson regression models to estimate the incidence rate ratio with 2015 quarter 4 as the reference.

For the retrospective study, we summarized patient characteristics, hospital characteristics, diagnoses potentially linked to staph bacteremia, and clinical outcome, including mortality rates, LOS, total cost, and 30-day readmission based on methicillin susceptibility and onset location (CO versus HO), employing the subset data. For the case-control study, we fit multivariable regression models to identify whether there was any difference in mortality, LOS, total cost, and 30-day readmission between those with and without staph bacteremia. The control group comprised individuals who had at least one blood culture but tested negative for staph bacteremia. We used Poisson regression for modeling LOS, gamma regression for total cost, and binomial regression for mortality and 30-day readmission. The models were adjusted for age, sex, ALaRMS,^20^ payor, number of staffed beds, hospital teaching status, and hospital urbanicity. All analyses were carried out using R software version 4.1.2 software (R Foundation for Statistical Computing, Vienna, Austria) with R Studio (Boston, MA).

## Results

**Figure 1.**
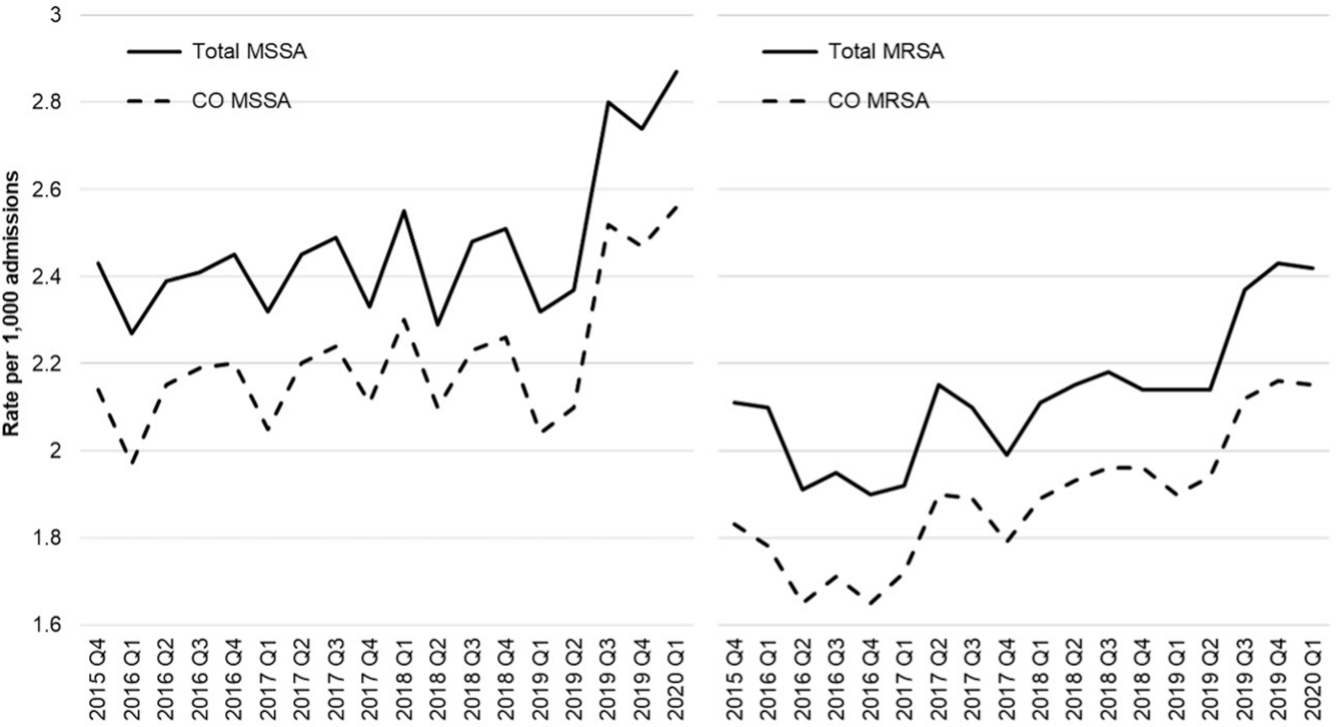
Trends of total and community-onset staph bacteremia (per 1,000 admissions) among 267 hospitals in the United States from 2015 to 2020. MSSA, methicillin-susceptible *Staphylococcus aureus*; MRSA, methicillin-resistant *Staphylococcus aureus*; CO, community-onset. P for trend: Total MSSA, <0.0006; CO MSSA, <0.0006; Total MRSA, < 0.0001; CO MRSA, < 0.0001.

**Figure 2.**
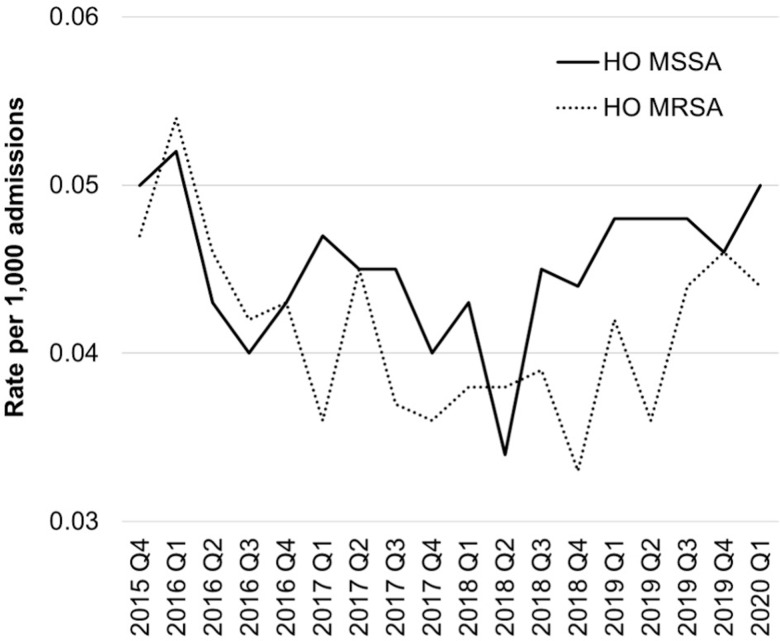
Trends of hospital-onset staph bacteremia (per 1,000 patient days at risk) among 267 hospitals in the United States from 2015 to 2020. MSSA, methicillin-susceptible *Staphylococcus aureus*; MRSA, methicillin-resistant *Staphylococcus aureus*; HO, hospital-onset. P for trend: HO MSSA, 0.3; HO MRSA: 0.4.

During the study period, we observed a total of 9,202,650 admissions across 267 acute-care hospitals. The incidence of total MSSA remained relatively stable and showed a significant increase, rising from 2.43 per 1,000 admissions in the fourth quarter of 2015 to 2.87 per 1,000 admissions in the first quarter of 2020 (rate ratio: 1.18, 95% CI (1.09, 1.29), 2020 Q1 versus 2015 Q4). The CO MSSA showed similar patterns as total MSSA. (P for trend <0.0006; see Figure 1; Supplemental Table 3). The incidence of CO MRSA exhibited a steady increase, rising from 1.84 per 1,000 admissions in the fourth quarter of 2015 to 2.16 per 1,000 admissions in the first quarter of 2020 (P for trend < 0.0001, Figure 1). The incidence of total MRSA followed similar patterns as CO MRSA. However, the incidence of HO MSSA and HO MRSA remained relatively stable (P for trend was 0.3 and 0.4, respectively, Figure 2).

During the same study period, data from the subset (41 acute-care hospitals) revealed a total of 645,315 admissions (Table 1). Within this subset, we recorded a total of 3,730 cases of staph bacteremia, with 2,066 (55.4%) classified as MSSA and 1,664 (44.6%) as MRSA. Among the 2,066 cases of MSSA, 1,829 (88.5%) were categorized as community-onset (CO) and 237 (11.5%) as hospital-onset (HO). Of the 1,664 cases of MRSA, 1,477 (88.8%) were CO, and 187 (11.2%) were HO. Among the six pre-defined infectious diseases potentially associated with staph bacteremia, the most frequent infectious disease diagnosis was skin and soft tissue infection for both MRSA and MSSA (27.3% in the MRSA group and 25.7% in the MSSA group, respectively), followed by musculoskeletal infection (26.6% and 24.9%). The mean LOS in days was 12.4 in the CO MRSA group, 24.8 in the HO MRSA group, 10.2 in the CO MSSA group, and 19.0 in the HO MSSA group. In-hospital mortality rates were 10.8% in the CO MRSA group, 25.1% in the HO MRSA group, 7.5% in the CO MSSA group, and 19.8% in the HO MSSA group. The total median costs were $20,300 in the CO MRSA group and $41,700 in the HO MRSA group, $17,300 in the CO MSSA group, and $33,400 in the HO MSSA group. The characteristics and clinical outcomes of the control group are shown in Table 1.

**Table 1.** Patient demographics, characteristics, and staph bacteremia-associated clinical outcomes from 41 acute-care hospitals in the United States between 2015 and 2022

	MRSA			MSSA			Control
	Overall	CO MRSA	HO MRSA	Overall	CO MSSA	HO MSSA	
	(N=1664)	(N=1477)	(N=187)	(N=2066)	(N=1829)	(N=237)	(N=198455)
**Hospitalization year**							
2015	59 (3.5%)	48 (3.2%)	11 (5.9%)	68 (3.3%)	59 (3.2%)	9 (3.8%)	6157 (3.1%)
2016	605 (36.4%)	532 (36.0%)	73 (39.0%)	729 (35.3%)	653 (35.7%)	76 (32.1%)	66633 (33.6%)
2017	693 (41.6%)	616 (41.7%)	77 (41.2%)	843 (40.8%)	746 (40.8%)	97 (40.9%)	82170 (41.4%)
2018	261 (15.7%)	237 (16.0%)	24 (12.8%)	357 (17.3%)	308 (16.8%)	49 (20.7%)	36689 (18.5%)
2019	46 (2.8%)	44 (3.0%)	2 (1.1%)	69 (3.3%)	63 (3.4%)	6 (2.5%)	6806 (3.4%)
**Patient characteristics**							
*Age*							
18–40	233 (14.0%)	206 (13.9%)	27 (14.4%)	283 (13.7%)	255 (13.9%)	28 (11.8%)	24085 (12.1%)
41–64	651 (39.1%)	580 (39.3%)	71 (38.0%)	927 (44.9%)	829 (45.3%)	98 (41.4%)	71559 (36.1%)
65–80	562 (33.8%)	493 (33.4%)	69 (36.9%)	615 (29.8%)	535 (29.3%)	80 (33.8%)	67389 (34.0%)
>80	218 (13.1%)	198 (13.4%)	20 (10.7%)	241 (11.7%)	210 (11.5%)	31 (13.1%)	35422 (17.8%)
Male	714 (42.9%)	623 (42.2%)	91 (48.7%)	800 (38.7%)	714 (39.0%)	86 (36.3%)	96155 (48.5%)
*ALaRMS score*							
Mean (SD)	59.9 (23.8)	60.8 (23.8)	53.0 (22.1)	57.3 (23.8)	57.8 (24.1)	53.6 (21.6)	50.1 (21.3)
Median [Q1, Q3]	59.0 [44.0, 75.0]	60.0 [44.8, 76.0]	51.5 [39.8, 64.3]	55.0 [42.0, 71.0]	55.0 [42.0, 73.0]	52.5 [40.0, 66.0]	48.0 [36.0, 63.0]
Missing	4 (0.2%)	1 (0.1%)	3 (1.6%)	5 (0.2%)	4 (0.2%)	1 (0.4%)	794 (0.4%)
*Payor class*							
Medicaid	272 (16.3%)	246 (16.7%)	26 (13.9%)	282 (13.6%)	246 (13.5%)	36 (15.2%)	124845 (62.9%)
Medicare	1047 (62.9%)	935 (63.3%)	112 (59.9%)	1141 (55.2%)	1008 (55.1%)	133 (56.1%)	20660 (10.4%)
Other	39 (2.3%)	34 (2.3%)	5 (2.7%)	61 (3.0%)	55 (3.0%)	6 (2.5%)	4271 (2.2%)
Private	233 (14.0%)	196 (13.3%)	37 (19.8%)	474 (22.9%)	426 (23.3%)	48 (20.3%)	39800 (20.1%)
Uninsured	71 (4.3%)	64 (4.3%)	7 (3.7%)	106 (5.1%)	92 (5.0%)	14 (5.9%)	8593 (4.3%)
Missing	2 (0.1%)	2 (0.1%)	0 (0%)	2 (0.1%)	2 (0.1%)	0 (0%)	286 (0.1%)
**Hospital characteristics**							
*Staffed bed size*							
<100	53 (3.2%)	44 (3.0%)	9 (4.8%)	69 (3.3%)	60 (3.3%)	9 (3.8%)	8744 (4.4%)
100–300	449 (27.0%)	415 (28.1%)	34 (18.2%)	567 (27.4%)	508 (27.8%)	59 (24.9%)	54469 (27.4%)
>300	1162 (69.8%)	1018 (68.9%)	144 (77.0%)	1430 (69.2%)	1261 (68.9%)	169 (71.3%)	135242 (68.1%)
Teaching hospital	1068 (64.2%)	942 (63.8%)	126 (67.4%)	1385 (67.0%)	1214 (66.4%)	171 (72.2%)	121819 (61.4%)
Urban hospital	1379 (82.9%)	1221 (82.7%)	158 (84.5%)	1753 (84.9%)	1545 (84.5%)	208 (87.8%)	162989 (82.1%)
*Census division*							
East North Central	362 (21.8%)	311 (21.1%)	51 (27.3%)	477 (23.1%)	420 (23.0%)	57 (24.1%)	41144 (20.7%)
East South Central	360 (21.6%)	316 (21.4%)	44 (23.5%)	236 (11.4%)	209 (11.4%)	27 (11.4%)	28962 (14.6%)
Middle Atlantic	74 (4.4%)	70 (4.7%)	4 (2.1%)	147 (7.1%)	142 (7.8%)	5 (2.1%)	14272 (7.2%)
Mountain	40 (2.4%)	39 (2.6%)	1 (0.5%)	82 (4.0%)	70 (3.8%)	12 (5.1%)	5496 (2.8%)
South Atlantic	64 (3.8%)	54 (3.7%)	10 (5.3%)	14 (0.7%)	10 (0.5%)	4 (1.7%)	7673 (3.9%)
West North Central	38 (2.3%)	35 (2.4%)	3 (1.6%)	68 (3.3%)	57 (3.1%)	11 (4.6%)	5210 (2.6%)
West South Central	726 (43.6%)	652 (44.1%)	74 (39.6%)	1042 (50.4%)	921 (50.4%)	121 (51.1%)	95698 (48.2%)
**Comorbidities**							
CLABSI	138 (8.3%)	117 (7.9%)	21 (11.2%)	154 (7.5%)	135 (7.4%)	19 (8.0%)	338 (0.2%)
Infective endocarditis	290 (17.4%)	264 (17.9%)	26 (13.9%)	349 (16.9%)	328 (17.9%)	21 (8.9%)	1895 (1.0%)
Musculoskeletal infections	443 (26.6%)	422 (28.6%)	21 (11.2%)	515 (24.9%)	496 (27.1%)	19 (8.0%)	8203 (4.1%)
Phlebitis and thrombophlebitis	23 (1.4%)	19 (1.3%)	4 (2.1%)	47 (2.3%)	24 (1.3%)	23 (9.7%)	680 (0.3%)
Pneumonia	392 (23.6%)	334 (22.6%)	58 (31.0%)	399 (19.3%)	352 (19.2%)	47 (19.8%)	43312 (21.8%)
Skin and soft tissue infections	455 (27.3%)	431 (29.2%)	24 (12.8%)	531 (25.7%)	495 (27.1%)	36 (15.2%)	23651 (11.9%)
**Outcomes**							
Readmission within 30 days	258 (15.5%)	222 (15.0%)	36 (19.3%)	325 (15.7%)	286 (15.6%)	39 (16.5%)	28545 (14.4%)
Expired at discharge	207 (12.4%)	160 (10.8%)	47 (25.1%)	185 (9.0%)	138 (7.5%)	47 (19.8%)	9753 (4.9%)
*LOS*							
Mean (SD)	13.8 (13.0)	12.4 (10.6)	24.8 (22.0)	11.2 (8.89)	10.2 (7.63)	19.0 (13.1)	7.25 (7.51)
Median [Q1, Q3]	10.0 [6.75, 17.0]	9.00 [6.00, 15.0]	19.0 [12.0, 29.0]	9.00 [6.00, 14.0]	8.00 [6.00, 12.0]	15.0 [11.0, 23.0]	5.00 [3.00, 8.00]
*Total cost*							
Mean (SD)	37700 (52500)	32600 (40800)	78400 (97700)	28200 (30800)	25300 (26500)	50900 (47700)	18800 (34500)
Median [Q1, Q3]	21600 [13000, 40800]	20300 [12400, 37200]	41700 [24400, 86500]	18800 [11900, 32200]	17300 [11400, 29300]	33400 [20500, 59500]	9770 [5670, 19000]
Ever ICU	697 (41.9%)	586 (39.7%)	111 (59.4%)	788 (38.1%)	655 (35.8%)	133 (56.1%)	56028 (28.2%)
*ICU LOS*							
Mean (SD)	7.40 (9.94)	6.46 (9.09)	12.4 (12.5)	6.03 (7.72)	5.19 (6.47)	10.2 (11.2)	5.18 (6.69)
Median [Q1, Q3]	4.28 [1.96, 9.09]	3.84 [1.84, 7.94]	8.52 [3.22, 17.1]	3.65 [1.63, 7.25]	3.18 [1.49, 6.15]	5.80 [2.68, 14.0]	2.98 [1.60, 6.12]
Missing	992 (59.6%)	912 (61.7%)	80 (42.8%)	1301 (63.0%)	1193 (65.2%)	108 (45.6%)	145290 (73.2%)

Note: MSSA, methicillin-susceptible *Staphylococcus aureus*; MRSA, methicillin-resistant *Staphylococcus aureus*; CO, community-onset; HO, hospital-onset; ALaRMs Score, Acute Laboratory Risk of Mortality Score; LOS, length of stay (days).

The model estimated and association between staph bacteremia and LOS, mortality, readmission, and total cost, stratified by methicillin susceptibility and onset of location, is summarized in Table 2. Compared to those without staph bacteremia, LOS was significantly longer in the CO MRSA group (10.0 days vs. 6.6 days in the control group, p < 0.0001) and in the HO MRSA group (17.9 days vs. 6.6 days, p < 0.001). Mortality was significantly higher in the CO MRSA group (4.0% vs. 2.9%, p < 0.001) and in the HO MRSA group (12.3% vs 2.9%, p < 0.0001) compared to the control group. Readmission was more common in the HO MRSA group (14% vs. 10%, p = 0.036) but similar in the CO MRSA group (10% vs 10%, p = 0.7105) compared to the control group. Total cost was significantly higher in the CO MRSA group ($24,437 vs. $15,261, p < 0.0001) and HO MRSA group ($45,444 vs. $15,261 p < 0.0001) compared to those without staph bacteremia. A similar trend was observed in the CO MSSA and HO MSSA groups compared to the control group, except for readmission; readmission was similar to those without MSSA.

**Table 2.** Association between MRSA/MSSA with length of stay, mortality, 30-day readmission, and total cost

	Control	CO	HO	Estimated difference between CO and control	Estimated difference between HO and control
**MRSA**					
Subgroup N	198,455	1,477	187		
LOS (days)	6.6 (6,7.4)	10 (9,11.1)	17.9 (16,20)	3.4‡	11.3‡
Mortality (%)	0.029 (0.02,0.04)	0.04 (0.027,0.057)	0.123 (0.078,0.19)	1.51†	4.32‡
Readmission (%)	0.1 (0.09,0.11)	0.1 (0.08,0.12)	0.14 (0.1,0.19)	1.39^ns^	1.4†
Total cost ($)	15261 (11901,19570)	24437 (19004,31424)	45444 (34727,59470)	9176‡	30183.0‡
**MSSA**					
Subgroup N	198,455	1,829	237		
LOS (days)	6.6 (6,7.4)	8.6 (7.7,9.5)	14.2 (12.8,15.8)	1.9‡	7.6‡
Mortality (%)	0.029 (0.02,0.04)	0.031 (0.021,0.045)	0.094 (0.059,0.146)	1.29^ns^	3.27‡
Readmission (%)	0.1 (0.09,0.11)	0.1 (0.09,0.12)	0.12 (0.08,0.16)	1.08^ns^	1.16^ns^
Total cost ($)	15239 (11250,20642)	20227 (14905,27449)	32738 (23821,44993)	4988‡	17499.2‡

ns , not statistically significant; †, <0.05; ‡<0.0001.

Note. MSSA, methicillin-susceptible *Staphylococcus aureus*; MRSA, methicillin-resistant *Staphylococcus aureus*; CO, community-onset; HO, hospital-onset; LOS, length of stay (days); ICU, intensive care unit. Full model: Outcomes ∼ MRSA/MSSA (CO/HO) and adjusted for year (2015–2017 vs. 2018–2019), sex, age, ever ICU status, ALaRMs score, bed size, teaching hospital status, urbanicity, and region.

## Discussion

This study revealed an increase in the incidence of MSSA and MRSA bacteremia between 2015 and 2020 across 267 acute-care hospitals in the United States. The primary driver behind this increase was CO bacteremia for both MSSA and MRSA. While HO MRSA demonstrated a decreasing trend from 2015 to 2018, as previously observed in similar studies, its incidence appeared to rebound in 2019 and 2020. Data obtained from a subset of 41 acute-care hospitals further highlighted that the most frequent infectious disease diagnosis associated with staph bacteremia was skin and soft tissue infection for patients with both MRSA and MSSA, followed by musculoskeletal infection. Compared to the control group, mortality, LOS, and total cost were all higher in those with staph bacteremia, regardless of methicillin susceptibility or onset location.

Staph bacteremia incidence is likely to vary based on the timing and locations of conducted studies, the risk factors prevalent in the studied population, and the infection control practices adopted in healthcare facilities.^10^ NHSN at the CDC systematically gathers healthcare-associated infection (HAI) data from a wide array of healthcare facilities in the United States.^24^ Nevertheless, the HAI report from NHSN exclusively covers CO and HO MRSA and does not encompass CO MSSA or HO MSSA. Although there are some efforts by EIP initiatives, with seven selected sites in the United States submitting data on invasive MSSA infections, including bacteremia, the true incidence and trend of staph bacteremia (both MSSA and MRSA) in the United States remain understudied.

This present study shows a significant increase in CO MSSA and CO MRSA between 2015 and 2020, with no significant changes observed in HO MSSA and HO MRSA. A similar trend has been observed in Europe.^25^ No observed increase in HO MSSA and MRSA bacteremia might be attributed to various infection control efforts, including improvements in preventing device- and procedure-associated infections, enhanced hand hygiene and environmental cleaning, admission screening with nasal swabs, and targeted ICU chlorhexidine-based decolonization practices.^26,27^ However, most of these efforts have primarily focused on MRSA in hospital settings. Therefore, new guidance may be needed for high-risk outpatients, such as those with peripherally inserted central catheters, ports, pacemakers, implantable cardioverter-defibrillators, hemodialysis catheters, and prosthetic joint replacement.^28,29^ Additionally, the ongoing opioid epidemic is likely contributing to the increased CO staph bacteremia.^30^ Although a recent study revealed that post-discharge MRSA decolonization with chlorhexidine and mupirocin led to a 30% lower risk of MRSA infection than education alone,^31^ there is currently no widely implemented approach for *S. aureus* carriers in outpatient settings, except for the recommendation of peri-operative decolonization and decolonization for individuals with recurrent staph infections.^32^

While the in-hospital mortality rate among those with staph bacteremia is high compared to controls, it is important to note that other delineations of bacteremia also exhibit disproportionate costs, LOS, and mortality outcomes.^33,34^ Notably, mortality was higher in HO bacteremia, reaching 24.1% for MRSA and 19.8% for MSSA compared to CO bacteremia. Our study confirmed that the incidence of HO staph bacteremia did not continuously decrease. A recent study across 123 Veterans Affairs (VA) healthcare systems, examining all hospital-acquired infections due to MRSA (including HO MRSA bacteremia), also indicated an increased incidence of MRSA infection.^35^ Consequently, HO staph bacteremia should remain a focus for public agencies, necessitating ongoing active surveillance and mitigation efforts.

This study has several limitations. First, we only used the results of the first blood culture to classify CO vs. HO bacteremia. Some patients developed HO bacteremia after being admitted with CO bacteremia but were categorized as having CO bacteremia in the present study. This could potentially lead to misclassification bias and an underestimation of HO bacteremia. However, only 1.1% of our cohort had subsequent positive cultures. Second, the necessary data for the retrospective cohort study were not available for all patients across 267 hospitals, so we used a subset of data from 41 hospitals, which could potentially introduce selection bias. Additionally, results may not be generalizable to the entire US population. Third, as is the case with any research relying on administrative data, we had to depend on the International Classification of Diseases, Tenth Revision, Clinical Modification (ICD-10-CM) coding for identifying six clinical diagnoses. Therefore, local coding practices could influence the accuracy of diagnostic coding, and bias cannot be ruled out. However, we used microbiological data to identify the population with true staph bacteremia. Fourth, the presence of orthopedic or cardiac prosthetic devices in the context of staph bacteremia increases the risk of relapsed infection. Although we included patients who presented with infections involving prosthetic devices, whether those devices were removed during treatment could not be determined. Moreover, we did not have access to information on the antibiotics used for treating staph bacteremia or whether source control was achieved when the source was identified. Fifth, due to the nonrandomized nature of the study, residual confounding in the outcome analyses cannot be ruled out. Sixth, we did not investigate which staph bacteremia was associated with intravenous drug use. Seventh, we did not have information regarding the infection control measures (e.g., contact precautions, admission screening) implemented by each hospital. Last, it is important to note that our study did not cover the period when the COVID-19 pandemic emerged in the United States, and trends might have changed in 2020.

In conclusion, the incidence of CO MSSA and CO MRSA increased between 2015 and 2020 across 267 acute-care hospitals in the United States, while HO MSSA and MRSA remained stable. In comparison to those without staph bacteremia, LOS, mortality, and total cost were higher in individuals with staph bacteremia, irrespective of methicillin susceptibility or onset location. In light of a potential HOB metric as proposed by the CDC, antibiograms produced at the hospital level may want to consider demarcating CO and HO resistance trends given that empirical and pre-op prophylaxis antimicrobial choices by clinicians and antimicrobial stewardship teams are often predicated on antibiogram information. Irrespective of a mandatory reporting requirement, the outcomes we describe may warrant further studies on the merits of tracking trends of both MRSA and MSSA and how to operationalize locally relevant resistance patterns into antimicrobial stewardship and infection prevention workflows.

## Supporting information

Kobayashi et al. supplementary material Kobayashi et al. supplementary material

## Supplementary material

For supplementary material accompanying this paper visit http://doi.org/10.1017/ash.2024.402. click here to view supplementary material

## References

[1] Rhee Y , Aroutcheva A , Hota B , Weinstein RA , Popovich KJ. Evolving epidemiology of Staphylococcus aureus Bacteremia. Infect Control Hosp Epidemiol 2015;36:1417–1422. 26372679 10.1017/ice.2015.213

[2] El Atrouni WI , Knoll BM , Lahr BD , Eckel-Passow JE , Sia IG , Baddour LM. Temporal trends in the incidence of Staphylococcus aureus bacteremia in Olmsted County, Minnesota, 1998 to 2005: a population-based study. Clin Infect Dis: An Official Publication of the Infect Dis Soc America. 2009;49:e130-8. 10.1086/648442 PMC3050712 19916797

[3] Chong YP , Moon SM , Bang KM , Park HJ , Park SY , Kim MN , et al. Treatment duration for uncomplicated Staphylococcus aureus bacteremia to prevent relapse: analysis of a prospective observational cohort study. Antimicrob Agents Chemother 2013;57:1150–1156. 23254436 10.1128/AAC.01021-12 PMC3591920

[4] Chang FY , MacDonald BB , Peacock JE, Jr., Musher DM , Triplett P , Mylotte JM , et al. A prospective multicenter study of Staphylococcus aureus bacteremia: incidence of endocarditis, risk factors for mortality, and clinical impact of methicillin resistance. Medicine 2003;82:322–332. 14530781 10.1097/01.md.0000091185.93122.40

[5] Tacconelli E , Carrara E , Savoldi A , Harbarth S , Mendelson M , Monnet DL , et al. Discovery, research, and development of new antibiotics: the WHO priority list of antibiotic-resistant bacteria and tuberculosis. Lancet Infect Dis 2018;18:318–327. 29276051 10.1016/S1473-3099(17)30753-3

[6] Kourtis AP , Hatfield K , Baggs J , Mu Y , See I , Epson E , et al. Vital signs: epidemiology and recent trends in methicillin-resistant and in methicillin-susceptible Staphylococcus aureus bloodstream infections - United States. MMWR Morbidity and mortality weekly report 2019;68:214–219. 30845118 10.15585/mmwr.mm6809e1 PMC6421967

[7] Sun H , Wei C , Liu B , Jing H , Feng Q , Tong Y , et al. Induction of systemic and mucosal immunity against methicillin-resistant Staphylococcus aureus infection by a novel nanoemulsion adjuvant vaccine. Int J Nanomedicine 2015;10:7275–7290. 26664118 10.2147/IJN.S91529 PMC4672755

[8] Wisplinghoff H , Bischoff T , Tallent SM , Seifert H , Wenzel RP , Edmond MB. Nosocomial bloodstream infections in US hospitals: analysis of 24,179 cases from a prospective nationwide surveillance study. Clin Infect Dis: An Official Publication of the Infect Dis Soc America 2004;39:309–317. 10.1086/421946 15306996

[9] Gagliotti C , Hogberg LD , Billstrom H , Eckmanns T , Giske CG , Heuer OE , et al. Staphylococcus aureus bloodstream infections: diverging trends of meticillin-resistant and meticillin-susceptible isolates, EU/EEA, 2005 to 2018. Euro Surveill 2021;26. 10.2807/1560-7917.ES.2021.26.46.2002094 PMC8603406 34794536

[10] Hindy JR , Quintero-Martinez JA , Lee AT , Scott CG , Gerberi DJ , Mahmood M , et al. Incidence trends and epidemiology of Staphylococcus aureus Bacteremia: a systematic review of population-based studies. Cureus 2022;14:e25460. 35774691 10.7759/cureus.25460 PMC9239286

[11] Mostofsky E , Lipsitch M , Regev-Yochay G. Is methicillin-resistant Staphylococcus aureus replacing methicillin-susceptible S. aureus? J Antimicrob Chemother 2011;66:2199–2214. 21737459 10.1093/jac/dkr278 PMC3172038

[12] de Kraker ME , Jarlier V , Monen JC , Heuer OE , van de Sande N , Grundmann H. The changing epidemiology of bacteraemias in Europe: trends from the European Antimicrobial Resistance Surveillance System. Clinical microbiology and infection : the official publication of the European Society of Clinical Microbiology and Infectious Diseases 2013;19:860–868. 23039210 10.1111/1469-0691.12028

[13] Klein EY , Mojica N , Jiang W , Cosgrove SE , Septimus E , Morgan DJ , Laxminarayan R. Trends in methicillin-resistant Staphylococcus aureus hospitalizations in the United States, 2010–2014. Clinical infectious diseases : an official publication of the Infectious Diseases Society of America 2017;65:1921–1923. 29020322 10.1093/cid/cix640

[14] Jones M , Jernigan JA , Evans ME , Roselle GA , Hatfield KM , Samore MH. Vital signs: trends in Staphylococcus aureus infections in veterans affairs medical centers - United States, 2005–2017. MMWR Morbidity and Mortality Weekly Report 2019;68:220–224. 30845116 10.15585/mmwr.mm6809e2 PMC6421970

[15] Hindy JR , Quintero-Martinez JA , Lahr BD , Palraj R , Go JR , Fida M , et al. Incidence of Monomicrobial Staphylococcus aureus Bacteremia: A Population-Based Study in Olmsted County, Minnesota-2006 to 2020. Open Forum Infect Dis 2022;9:ofac190. 10.1093/ofid/ofac190 PMC9251673 35794939

[16] Centers for Disease Control and Prevention. National Healthcare Safety Network (NHSN). NHSNCoLab. Available at https://www.cdc.gov/nhsn/nhsncolab/index.html.

[17] Kaye KS , Gupta V , Mulgirigama A , Joshi AV , Scangarella-Oman NE , Yu K , et al. Antimicrobial resistance trends in urine Escherichia coli isolates from adult and adolescent females in the United States From 2011 to 2019: rising ESBL strains and impact on patient management. Clinical infectious diseases : an official publication of the Infectious Diseases Society of America 2021;73:1992–1999. 34143881 10.1093/cid/ciab560 PMC8664433

[18] Yu KC , Yamaga C , Vankeepuram L , Tabak YP. Relationships between creatinine increase and mortality rates in patients given vancomycin in 76 hospitals: the increasing role of infectious disease pharmacists. Am J Health Syst Pharm 2021;78:2116–2125. 34125896 10.1093/ajhp/zxab247

[19] Yu KC , Ye G , Edwards JR , Gupta V , Benin AL , Ai C , Dantes R. Hospital-onset bacteremia and fungemia: an evaluation of predictors and feasibility of benchmarking comparing two risk-adjusted models among 267 hospitals. Infect Control Hosp Epidemiol 2022;43:1317–1325. 36082774 10.1017/ice.2022.211 PMC9588439

[20] Tabak YP , Sun X , Nunez CM , Johannes RS. Using electronic health record data to develop inpatient mortality predictive model: Acute Laboratory Risk of Mortality Score (ALaRMS). J Am Med Inform Assoc 2014;21:455–463. 24097807 10.1136/amiajnl-2013-001790 PMC3994855

[21] Centers for Disease Control and Prevention. Health, United States, 2020–2021. Geographic division or region. Available at https://www.cdc.gov/nchs/hus/sources-definitions/geographic-region.htm.

[22] Inagaki K , Weinberg JB , Kaul DR. Risk of Staphylococcus aureus Bacteremia before and after solid organ transplantation. Transplantation 2023;107:1820–1827. 36959162 10.1097/TP.0000000000004590

[23] WWAMI Rural Health Research Center. Rural urban commuting area codes. Available at https://depts.washington.edu/uwruca/.

[24] Centers for Disease Control and Prevention. National Healthcare Safety Network (NHSN). Available at https://www.cdc.gov/nhsn/index.html.

[25] Renggli L , Gasser M , Buetti N , Kronenberg A , Swiss Centre for Antibiotic R. Increase in methicillin-susceptible Staphylococcus aureus bloodstream infections in Switzerland: a nationwide surveillance study (2008–2021). Infection 2023;51:1025–1031. 36732413 10.1007/s15010-023-01980-6 PMC10352440

[26] Popovich KJ , Aureden K , Ham DC , Harris AD , Hessels AJ , Huang SS , et al. SHEA/IDSA/APIC Practice Recommendation: Strategies to prevent methicillin-resistant Staphylococcus aureus transmission and infection in acute-care hospitals: 2022 Update. Infect Control Hosp Epidemiol 2023;44:1039–1067. 37381690 10.1017/ice.2023.102 PMC10369222

[27] Lee BY , Bartsch SM , Wong KF , McKinnell JA , Cui E , Cao C , et al. Beyond the Intensive Care Unit (ICU): countywide impact of universal ICU Staphylococcus aureus decolonization. Am J Epidemiol 2016;183:480–489. 26872710 10.1093/aje/kww008 PMC4772440

[28] Souli M , Ruffin F , Choi SH , Park LP , Gao S , Lent NC , et al. Changing characteristics of Staphylococcus aureus Bacteremia: results from a 21-year, prospective, longitudinal study. Clinical infectious diseases : an official publication of the Infectious Diseases Society of America 2019;69:1868–1877. 31001618 10.1093/cid/ciz112 PMC6853684

[29] Rha B , See I , Dunham L , Kutty PK , Moccia L , Apata IW , et al. Vital Signs: Health Disparities in Hemodialysis-Associated Staphylococcus aureus Bloodstream Infections - United States, 2017–2020. MMWR Morbidity and mortality weekly report 2023;72:153–159. 36757874 10.15585/mmwr.mm7206e1 PMC9925139

[30] Quagliarello B , Cespedes C , Miller M , Toro A , Vavagiakis P , Klein RS , Lowy FD. Strains of Staphylococcus aureus obtained from drug-use networks are closely linked. Clinical infectious diseases : an official publication of the Infectious Diseases Society of America 2002;35(6):671–677. 12203163 10.1086/342196

[31] Huang SS , Singh R , McKinnell JA , Park S , Gombosev A , Eells SJ , et al. Decolonization to reduce postdischarge infection risk among MRSA carriers. N Engl J Med 2019;380:638–650. 30763195 10.1056/NEJMoa1716771 PMC6475519

[32] Bebko SP , Green DM , Awad SS. Effect of a preoperative decontamination protocol on surgical site infections in patients undergoing elective orthopedic surgery with hardware implantation. JAMA Surg 2015;150:390–395. 25738898 10.1001/jamasurg.2014.3480

[33] Yu KC , Jung M , Ai C. Characteristics , costs, and outcomes associated with central-line-associated bloodstream infection and hospital-onset bacteremia and fungemia in US hospitals. Infect Control Hosp Epidemiol 2023;44:1920–1926. 37424226 10.1017/ice.2023.132 PMC10755163

[34] Diekema DJ , Hsueh PR , Mendes RE , Pfaller MA , Rolston KV , Sader HS , Jones RN. The microbiology of bloodstream infection: 20-year trends from the SENTRY antimicrobial surveillance program. Antimicrob Agents Chemother 2019;63. 10.1128/AAC.00355-19 PMC6591610 31010862

[35] Evans ME , Simbartl LA , McCauley BP , Flarida LK , Jones MM , Harris AD , et al. Active surveillance and contact precautions for preventing methicillin-resistant Staphylococcus aureus healthcare-associated infections during the COVID-19 pandemic. Clinical infectious diseases : an official publication of the Infectious Diseases Society of America. 2023;77:1381–1386. 37390613 10.1093/cid/ciad388

