# Supplementary material for: Trends and outcomes in community-onset and hospital-onset Staphylococcus bacteremia among hospitals in the United States from 2015 to 2020
Source: Antimicrob Steward Healthc Epidemiol. 2024 Sep 16;4(1):e136. doi: 10.1017/ash.2024.402 (PMC11406565; doi:10.1017/ash.2024.402)
Supplement: Kobayashi et al. supplementary material [file S2732494X24004029sup001.docx]

**Supplemental Table 1a:** Demographics distribution of 267 acute care hospitals

| **Demographics** | **Category** | **Number of sites** | **%Total** |
| --- | --- | --- | --- |
| Total hospital | Total hospital | 267 |  |
| Bed size | <100 | 91 | 34.1% |
|  | 100-300 | 113 | 42.3% |
|  | >300 | 63 | 23.6% |
| Census division | East North Central | 48 | 18.0% |
|  | East South Central | 36 | 13.5% |
|  | Middle Atlantic | 40 | 15.0% |
|  | Mountain | 10 | 3.7% |
|  | New England | 4 | 1.5% |
|  | Pacific | 23 | 8.6% |
|  | South Atlantic | 45 | 16.9% |
|  | West North Central | 9 | 3.4% |
|  | West South Central | 52 | 19.5% |
| Teaching status | Non-Teaching | 161 | 60.3% |
|  | Teaching | 106 | 39.7% |
| Urbanicity | Rural | 100 | 37.5% |
|  | Urban | 167 | 62.5% |

**Supplemental Table 1b:** Demographics distribution of 41 acute care hospitals

| **Demographics** | **Category** | **Number of sites** | **%Total** |
| --- | --- | --- | --- |
| Total hospital | Total hospital | 41 |  |
| Bed size | <100 | 3 | 7% |
|  | 100-300 | 18 | 44% |
|  | >300 | 20 | 49% |
| Census division | East North Central | 14 | 34% |
|  | East South Central | 5 | 12% |
|  | Middle Atlantic | 2 | 5% |
|  | Mountain | 2 | 5% |
|  | New England | 0 | 0% |
|  | Pacific | 0 | 0% |
|  | South Atlantic | 2 | 5% |
|  | West North Central | 2 | 5% |
|  | West South Central | 14 | 34% |
| Teaching status | Non-Teaching | 17 | 41% |
|  | Teaching | 24 | 59% |
| Urbanicity | Rural | 9 | 22% |
|  | Urban | 32 | 78% |

**Supplemental Table 2: ICD 10 codes for identifying infections during the hospitalization**

| **ICD 10 Description** | **ICD 10 Code** |
| --- | --- |
| Musculoskeletal infections (osteomyelitis, septic arthritis, pyomyositis, and intraspinal abscess), including those involving prosthesis | M00, M600, M462, M86, M845, T846, T847, G061 |
| Skin and soft tissue infections | L00-L089 |
| Pneumonia | J15, J15.21, J15.8-J16, J16.8-J18.9, J85.1-J85.2, J86 |
| Catheter associated blood stream infection | T80.2X |
| Infective endocarditis, including those involving prosthesis | I33, I38, I39, T82.6, T82.7 |
| Phlebitis and thrombophlebitis of unspecified site | I80.XX |

**Supplemental Table 3:** Incidence rate ratio of MRSA/MSSA by total, CO, and HO, Year 2015 Q4 to 2022 Q1, 267 hospitals

|  | **Rate ratio (95% CI)** | | | | | |
| --- | --- | --- | --- | --- | --- | --- |
| **Year Quarter** | **Total MRSA** | **CO MRSA** | **HO MRSA** | **Total MSSA** | **CO MSSA** | **HO MSSA** |
| 2015 Q4 | 1.0 (Ref) | 1.0 (Ref) | 1.0 (Ref) | 1.0 (Ref) | 1.0 (Ref) | 1.0 (Ref) |
| 2016 Q1 | 1.00 (0.90, 1.10) | 0.97 (0.87, 1.08) | 1.16 (0.88, 1.51) | 0.94 (0.85, 1.03) | 0.92 (0.83, 1.02) | 1.05 (0.80, 1.37) |
| 2016 Q2 | 0.91 (0.82, 1.00) | 0.90 (0.81, 1.00) | 0.98 (0.75, 1.30) | 0.99 (0.90, 1.08) | 1.00 (0.91, 1.11) | 0.86 (0.65, 1.13) |
| 2016 Q3 | 0.93 (0.84, 1.02) | 0.94 (0.84, 1.04) | 0.90 (0.68, 1.18) | 1.00 (0.91, 1.09) | 1.02 (0.93, 1.13) | 0.80 (0.60, 1.05) |
| 2016 Q4 | 0.90 (0.82, 0.99) | 0.90 (0.81, 1.00) | 0.91 (0.69, 1.20) | 1.01 (0.92, 1.10) | 1.03 (0.94, 1.13) | 0.87 (0.66, 1.13) |
| 2017 Q1 | 0.91 (0.83, 1.01) | 0.94 (0.84, 1.04) | 0.77 (0.58, 1.01) | 0.96 (0.88, 1.05) | 0.96 (0.87, 1.05) | 0.96 (0.74, 1.24) |
| 2017 Q2 | 1.02 (0.93, 1.12) | 1.03 (0.94, 1.14) | 0.96 (0.73, 1.25) | 1.01 (0.93, 1.10) | 1.03 (0.94, 1.13) | 0.90 (0.69, 1.17) |
| 2017 Q3 | 1.00 (0.91, 1.10) | 1.03 (0.93, 1.14) | 0.79 (0.60, 1.04) | 1.03 (0.94, 1.12) | 1.05 (0.95, 1.15) | 0.90 (0.70, 1.17) |
| 2017 Q4 | 0.95 (0.86, 1.04) | 0.98 (0.88, 1.08) | 0.77 (0.58, 1.01) | 0.96 (0.88, 1.05) | 0.99 (0.90, 1.08) | 0.80 (0.62, 1.05) |
| 2018 Q1 | 1.00 (0.91, 1.10) | 1.03 (0.93, 1.14) | 0.80 (0.61, 1.05) | 1.05 (0.96, 1.14) | 1.07 (0.98, 1.17) | 0.87 (0.67, 1.12) |
| 2018 Q2 | 1.02 (0.93, 1.12) | 1.05 (0.96, 1.16) | 0.80 (0.61, 1.05) | 0.94 (0.87, 1.03) | 0.98 (0.90, 1.08) | 0.68 (0.52, 0.89) |
| 2018 Q3 | 1.03 (0.94, 1.13) | 1.07 (0.97, 1.18) | 0.83 (0.64, 1.09) | 1.02 (0.94, 1.11) | 1.04 (0.95, 1.14) | 0.90 (0.70, 1.17) |
| 2018 Q4 | 1.02 (0.93, 1.12) | 1.07 (0.97, 1.18) | 0.70 (0.53, 0.92) | 1.03 (0.95, 1.13) | 1.06 (0.97, 1.16) | 0.88 (0.68, 1.13) |
| 2019 Q1 | 1.02 (0.93, 1.12) | 1.04 (0.94, 1.14) | 0.89 (0.68, 1.15) | 0.96 (0.88, 1.04) | 0.96 (0.87, 1.05) | 0.96 (0.75, 1.23) |
| 2019 Q2 | 1.02 (0.93, 1.12) | 1.06 (0.96, 1.17) | 0.76 (0.58, 1.00) | 0.98 (0.90, 1.07) | 0.98 (0.90, 1.07) | 0.97 (0.76, 1.25) |
| 2019 Q3 | 1.13 (1.03, 1.23) | 1.15 (1.05, 1.27) | 0.95 (0.73, 1.23) | 1.15 (1.06, 1.25) | 1.18 (1.08, 1.29) | 0.98 (0.76, 1.26) |
| 2019 Q4 | 1.15 (1.05, 1.26) | 1.18 (1.07, 1.30) | 0.98 (0.75, 1.27) | 1.13 (1.04, 1.23) | 1.16 (1.06, 1.27) | 0.93 (0.72, 1.20) |
| 2020 Q1 | 1.15 (1.05, 1.26) | 1.17 (1.06, 1.29) | 0.93 (0.72, 1.21) | 1.18 (1.09, 1.29) | 1.20 (1.10, 1.31) | 1.01 (0.79, 1.30) |

Abbreviations: MSSA, methicillin susceptible staphylococcus aureus; MRSA, methicillin resistant staphylococcus aureus; CO, community-onset; HO, hospital-onset; Ref, reference

P for trend: Total MSSA, <0.0006; CO MSSA, <0.0006; Total MRSA, < 0.0001; CO MRSA, < 0.0001; HO MSSA, 0.3; HO MRSA: 0.4
